# Delayed symptomatic intracerebral hemorrhages in neuro-Behcet’s disease

**DOI:** 10.1007/s00415-016-8363-1

**Published:** 2016-12-20

**Authors:** Woo-Jin Lee, Changwoon Choi, Jeong-Min Kim, Keun-Hwa Jung, Jae-Kyu Roh

**Affiliations:** 1 The Headquarter of the Army, Ministry of National Defence, Seoul, South Korea; 2 Program in Neuroscience, Neuroscience Research Institute of SNUMRC, College of Medicine, Seoul National University, Seoul, South Korea; 3 Department of Neurology, Korea University Guro Hospital, Seoul, South Korea; 4 Department of Neurology, Chung-Ang University Hospital, Seoul, South Korea; 5 Department of Neurology, Seoul National University Hospital, Seoul, South Korea; 6 Department of Neurology, The Armed Forces Capital Hospital, Bundang-gu, Yul-dong, Mountain 13-4, Sungnam, 463-040 South Korea

Dear Sirs,

Central nervous system involvement occurs in 10–30% of Behcet’s disease (BD). Although its symptoms gradually resolve without leaving significant sequelae in most cases, neurologic involvement of BD (NBD) can have progressive courses in some occasions, which are associated with poor neurologic prognosis and warrant stepping up the immune-modulating treatment [1, 4, 7]. However, we experienced a rare case of NBD which had a neurologic aggravation at subacute stages accompanied with delayed development of hemorrhages, which mimicked a true progression of disease.

A 50-year-old man complained of sudden onset left arm weakness recognized on waking-up. From 5 years before the event, he had experienced recurrent oral ulcers, skin rash, and arthritis. He had been diagnosed as BD [4, 7] and prescribed oral colchicine, which he arbitrarily stopped. At admission, initial brain MRI revealed hyper-signal intensities at right basal ganglia and centrum semiovale on diffusion-weighted images (DWI) and fluid-attenuated inversion recovery (FLAIR) sequences, without hypo-signal intensities on apparent diffusion coefficient (ADC) images, indicating a vasogenic edema as the main mechanism. No hemorrhagic lesion was detected in gradient-echo images (GRE). Magnetic resonance angiography, venography, and laboratory findings including serum d-dimer and coagulation profiles revealed no abnormality. After seven days from the onset, the patient reported worsening of left arm weakness. Follow-up MRI revealed a newly developed small hemorrhage at right basal ganglia with slightly increased extent of preexisting edema (Fig. 1). The left arm weakness lasted for a month before being completely resolved.

**Fig. 1:**
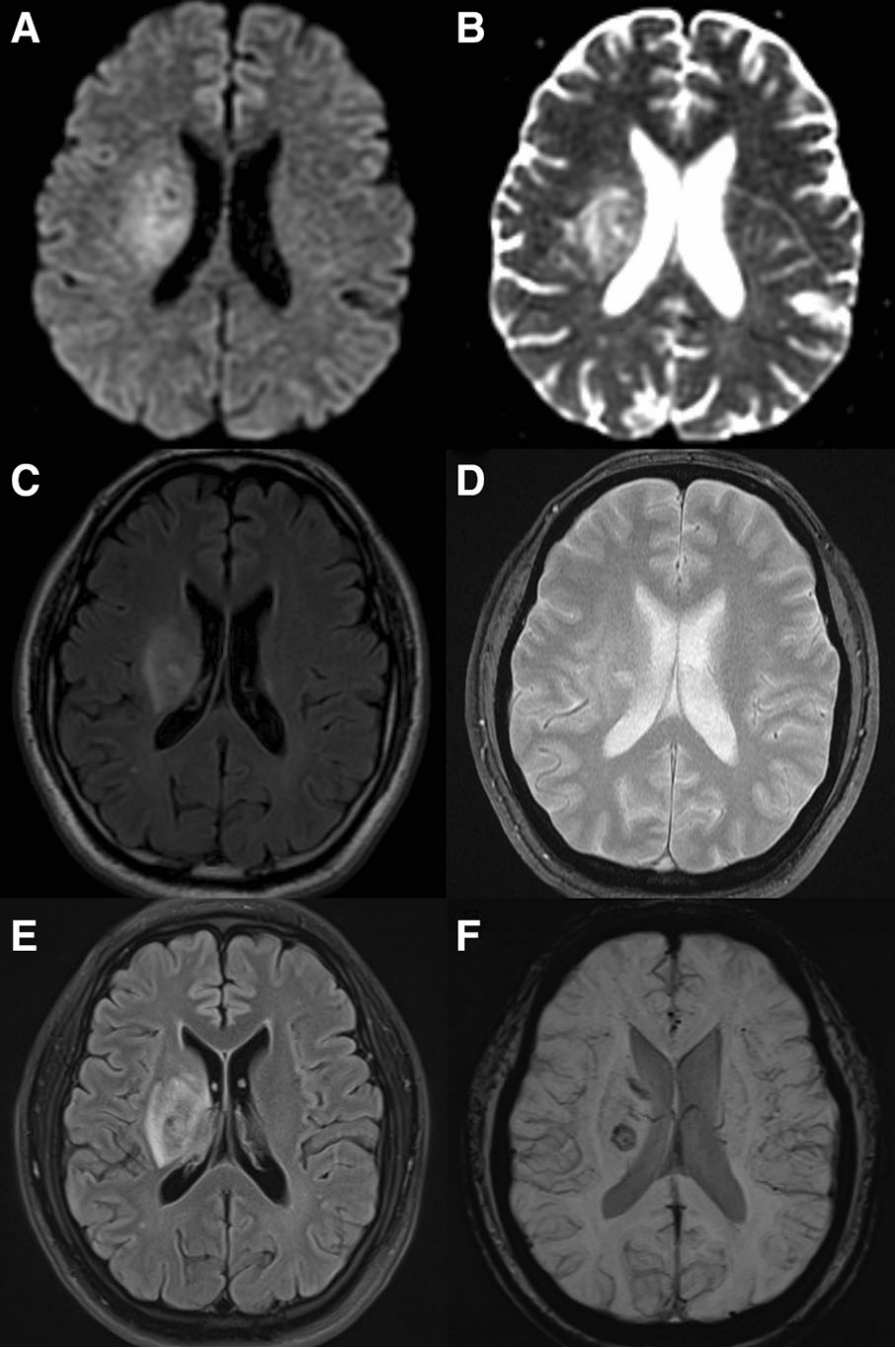
Initial and follow-up magnetic resonance images (MRI) at the first event. The initial MRI shows high signal intensity on diffusion-weighted image (DWI, **a**), without low signal intensity on apparent diffusion coefficient (ADC, **b**). Fluid-attenuated inversion recovery (FLAIR, **c**) images reveal increased signal intensities in right basal ganglia and centrum semiovale. No hemorrhagic lesion was detected in gradient-echo images (GRE, **d**). The follow-up MRI shows slightly increased extent of FLAIR high signal intensity area (**e**) and development of focal hemorrhage at right basal ganglia (**f**), compared to the initial MRI images

Six months later, he again developed a sudden weakness of both leg and dysarthria. On brain MRI, multiple hyper-signal intensities in right pons and bilateral centrum semiovale were found on DWI, without a hemorrhage on GRE. Similar to the previous attack, delayed progression of symptoms was reported after twelve days from the onset, related to the interval development of multiple hemorrhages in the affected areas on follow-up MRI (Fig. 2). The symptoms spontaneously resolved after a month.

**Fig. 2:**
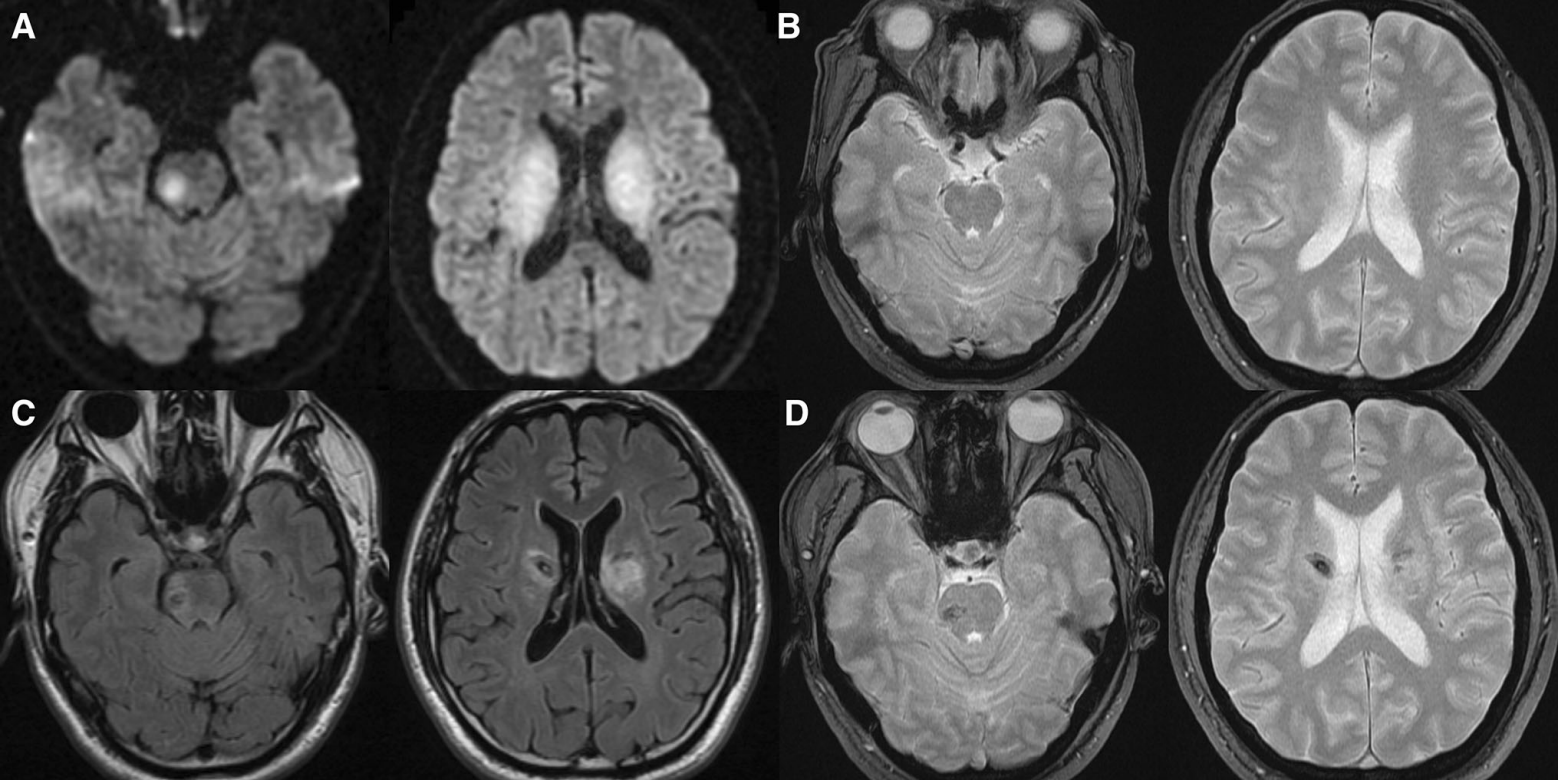
Initial and follow-up magnetic resonance images (MRI) at the second event. The initial MRI (**a**, **b**) shows high signal intensities in right pons and both centrum semiovale in diffusion-weighted image (DWI, **a**), without hemorrhagic lesion in gradient-echo (GRE, **b**). The follow-up MRI (**c**, **d**) shows decreased extent of edema on fluid-attenuated inversion recovery (FLAIR) images (**c**) and interval development of multiple hemorrhages on GRE (**d**) in the affected areas

Given that vasogenic edema might be responsible for the initial manifestation in both events, and that NBD frequently involves territories such as brainstem, spinal cord, and deep cerebral hemispheres [1, 2, 5, 7], a BD-associated venular-dominant small vessel vasculitis might be the underlying pathomechanism of the events [3, 4]. Furthermore, transient progressions of neurologic deficits at subacute stages might be due to slow and sustained extravasation of blood from a damaged small vessel [3, 9]. Hemorrhages in venular vasculitis are reported in the pulmonary, mucocutaneous involvement of BD [3, 9], and other types of systemic small vessel vasculitis [6]. Regarding NBD, while a large observational study of 200 patients did not report any hemorrhagic manifestations [1], one report described two cases of hematomas detected in peripheral lesion at subacute phases [8]. However, the clinical significance of a delayed hematoma development in NBD has not been demonstrated.

Although not yet elucidated, two main pathomechanistic factors might be involved in the delayed hematoma development in NBD: (1) increased intravascular pressure provoked by thrombotic occlusion of the proximal venules and (2) sustained extravasation of red blood cells via the disrupted walls of small vessels [3, 9]. This is distinct from sudden and rapid neurologic deteriorations in arterial origin hemorrhages or hemorrhagic transformation of arterial infarctions, which occurs in acute phases and is mediated by gushing out blood from damaged vessels.

As in this case, hemorrhages in NBD might have a significant clinical implication, by slow expansion of hemorrhages inducing a delayed and transient progression of neurologic symptoms. Notably, this should be distinguished from a true progression of NBD-associated vasculitic process in CNS, which requires urgent augmentation of immunotherapies. Follow-up MRI evaluation with susceptibility-weighted or gradient-echo images combined with FLAIR and DWI sequences can help the differential diagnosis.

## References

[1] Akman-Demir G, Serdaroglu P, Tasçi B (1999). Clinical patterns of neurological involvement in Behçet’s disease: evaluation of 200 patients. Brain.

[2] Al Kawi MZ, Bohlega S, Banna M (1991). MRI findings in neuro-Behcet’s disease. Neurology.

[3] Ehrlich GE (1997). Vasculitis in Behçet’s disease. Int Rev Immunol.

[4] Haghighi AB, Pourmand R, Nikseresht A-R (2005). Neuro-Behcet disease: a review. Neurologist.

[5] Haghighi AB, Sarhadi S, Farahangiz S (2011). MRI findings of neuro-Behcet’s disease. Clin Rheumatol.

[6] Jennette JC, Falk RJ (1997). Small-vessel vasculitis. N Engl J Med.

[7] Kidd D, Steuer A, Denman A, Rudge P (1999). Neurological complications in Behçet’s syndrome. Brain.

[8] Kikuchi S, Niino M, Shinpo K, Terae S, Tashiro K (2002). Intracranial hemorrhage in neuro-Behcet’s syndrome. Intern Med.

[9] Lakhanpal S, Tani K, Lie J, Katoh K, Ishigatsubo Y, Ohokubo T (1985). Pathologic features of Behçet’s syndrome: a review of Japanese autopsy registry data. Hum Pathol.

